# Time-dependent communication between multiple amino acids during protein folding†

**DOI:** 10.1039/d0sc07025d

**Published:** 2021-03-24

**Authors:** Song-Ho Chong, Sihyun Ham

**Affiliations:** Department of Chemistry, The Research Institute of Natural Sciences, Sookmyung Women's University Cheongpa-ro-47-gil 100, Yongsan-ku Seoul 04310 Korea sihyun@sookmyung.ac.kr

## Abstract

Cooperativity is considered to be a key organizing principle behind biomolecular assembly, recognition and folding. However, it has remained very challenging to quantitatively characterize how cooperative processes occur on a concerted, multiple-interaction basis. Here, we address how and when the folding process is cooperative on a molecular scale. To this end, we analyze multipoint time-correlation functions probing time-dependent communication between multiple amino acids, which were computed from long folding simulation trajectories. We find that the simultaneous multiple amino-acid contact formation, which is absent in the unfolded state, starts to develop only upon entering the folding transition path. Interestingly, the transition state, whose presence is connected to the macrostate cooperative behavior known as the two-state folding, can be identified as the state in which the amino-acid cooperativity is maximal. Thus, our work not only provides a new mechanistic view on how protein folding proceeds on a multiple-interaction basis, but also offers a conceptually novel characterization of the folding transition state and the molecular origin of the phenomenological cooperative folding behavior. Moreover, the multipoint correlation function approach adopted here is general and can be used to expand the understanding of cooperative processes in complex chemical and biomolecular systems.

## Abstract 2

Cooperativity in contact formation among multiple amino acids starts to develop upon entering the folding transition path and attains a maximum at the folding transition state, providing the molecular origin of the two-state folding behavior.

## Graphical abstract

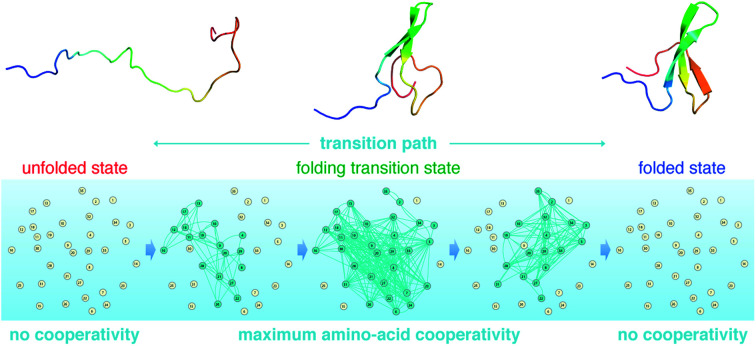

## Introduction

Biomolecular assembly, recognition and folding are complex processes in which building blocks, such as amino acids in proteins, search for favorable inter- or intra-molecular interactions in intricate manners.^1–3^ Cooperativity has been recognized to be a key concept associated with these processes.^4–6^ However, cooperativity in macromolecular systems is typically described at a phenomenological, macrostate level, and is broadly defined as a characteristic of processes in which intermediate states are disfavored, *i.e.*, only the extreme states are significantly populated. Such all-or-none behavior, corresponding to switching between “on” and “off” states, is critical in regulation and signaling to avoid undesirable effects. The all-or-none character in ligand binding—receptor binding sites are either empty or fully occupied—is the basis for the Hill equation, which provides a commonly adopted measure of cooperativity.^7^ The cooperativity concept in protein folding was also introduced at the macrostate level,^8^ conveying that folding proceeds in a two-state, all-or-none fashion.

Such a macrostate cooperativity concept, however, does not reveal underlying molecular mechanisms. In this regard, we notice that the cooperativity between two events *A* and *B* can in general be captured by the correlation, *χ* = *P*(*A*, *B*) – *P*(*A*)*P*(*B*), defined in terms of the joint probability *P*(*A*, *B*) and the product *P*(*A*)*P*(*B*) of the probabilities of individual events:^9,10^*χ* > 0 or *χ* < 0 corresponds to positive or negative cooperativity, respectively. For example, when *A* and *B* refer to ligand binding events to receptor sites *i* and *j*, *χ* > 0 indicates that the conditional probability *P*(*B*/*A*) = *P*(*A*, *B*)/*P*(*A*) is larger than *P*(*B*), *i.e.*, the ligand binding to site *i* enhances the binding affinity to site *j* from what it would be in isolation. Thus, the cooperativity formulated with *χ* is able to uncover the existence of a certain communication between molecular events occurring at distinct sites (the term “communication” is used here only in this sense, *i.e.*, when the correlation or cooperativity quantified by *χ* ≠ 0 is present). Owing to the recent advances in experimental and computational technologies, the folding transition path that was previously inaccessible has now become within our reach.^11–16^ The folding transition path is a small fraction of equilibrium folding trajectories where the folding process actually takes place. The transition path thus contains, in principle, all the mechanisms of protein folding, and there must be certain concerted molecular processes that underlie the macrostate folding cooperativity.

**Fig. 1:**
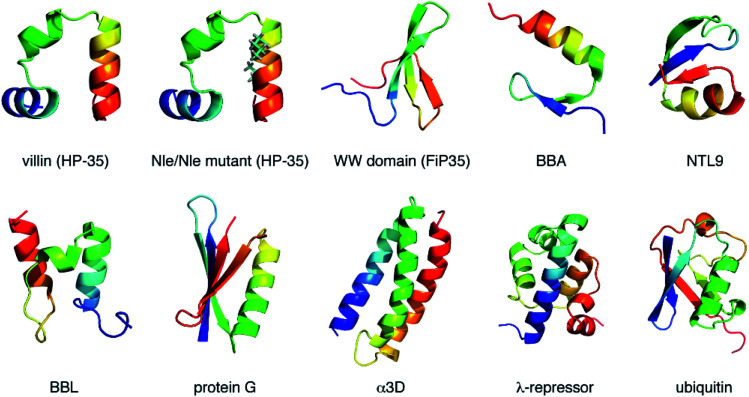
Proteins studied in the present work. Proteins are color coded according to the sequence, ranging from blue (N-terminus) to red (C-terminus). Norleucine (Nle) residues in the Nle/Nle mutant are indicated by the stick representations.

Here, we investigate the folding cooperativity through the correlation *χ* defined with microscopic events occurring in the transition path. This is done for a number of small globular proteins displayed in Fig. 1 (see also Table S1†), whose all-atom simulations were reported by Shaw and coworkers.^17–20^ Since protein folding requires the establishment of native amino-acid contacts, we will choose the formations of those contacts as the relevant microscopic events. Of particular interest in the present work is the timing (early, intermediate, or late stage) at which the cooperativity sets in during the transition path. To achieve this goal, *χ*(*t*) carrying the time-dependence shall be introduced, which hence probes time-dependent cooperativity or communication between amino acids. Thereby, we would like to address how and when the folding process is cooperative on a molecular scale. We will then argue how such microscopic cooperativity is connected to the emergence of the macrostate cooperative folding behavior.

## Results

**Fig. 2:**
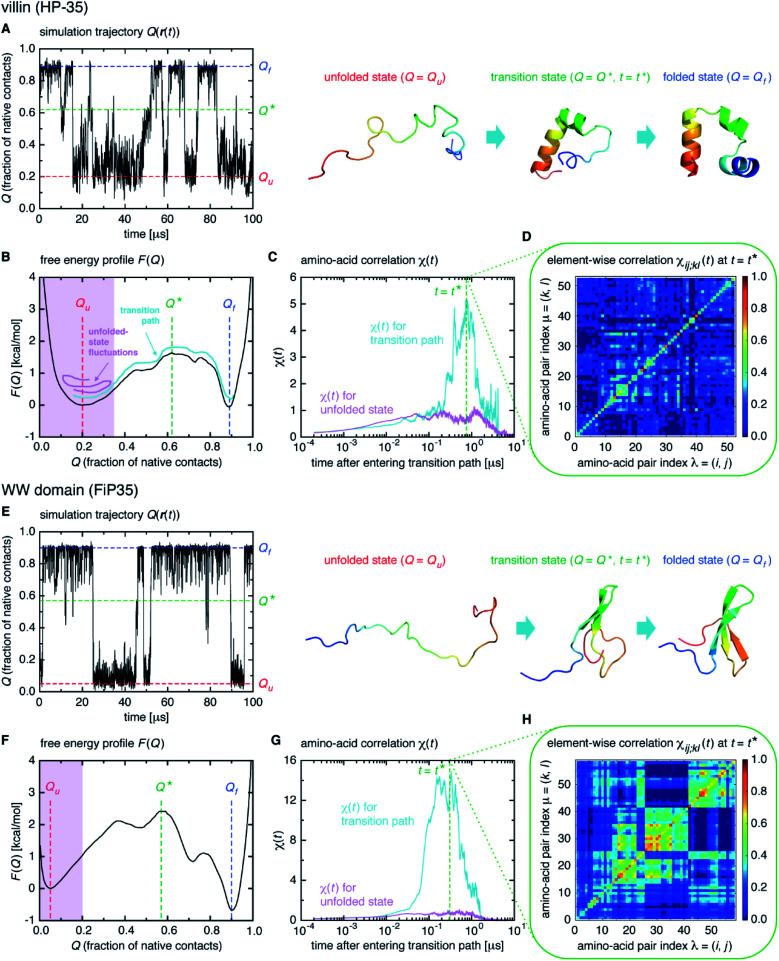
Time-dependent cooperativity between multiple amino acids. (A) Fraction of the native amino-acid contacts *Q*(***r***(*t*)) for the protein configuration ***r***(*t*) at time *t* for a 100 μs portion of the simulation trajectory of the villin headpiece subdomain (HP-35). (B) Folding free energy profile *F*(*Q*) *versus Q*. (C) *χ*(*t*) for the transition path (colored cyan) and for the unfolded state (colored magenta) on a logarithmic timescale. (D) Element-wise *χ*_*ij*;*kl*_(*t*) at *t* = t*. (E–H) Corresponding results for the WW domain (FiP35).

We start from surveying the folding behavior of the systems studied here. To succinctly describe our results, we will mainly deal with the α-helical villin headpiece subdomain (HP-35) in the following; the results for the β-sheet WW domain (FiP35) are also included in the main text, and those for the other eight systems are presented in Fig. S1 to S8.† The folding process is monitored by the fraction of native amino-acid contacts *Q* (0 ≤ *Q* ≤ 1), which was reported to be a good reaction coordinate of folding.^21^ We computed *Q*(***r***(*t*)) for each protein configuration ***r***(*t*) along the trajectory (Fig. 2A), and constructed the probability distribution *P*(*Q*) of sampled *Q*(***r***(*t*)) values. The folding reaction free energy profile is then obtained from *F*(*Q*) = −*k*_B_*T* log *P*(*Q*) with Boltzmann's constant *k*_B_ and temperature *T* (Fig. 2B). It is observed that the system stays most of the time either in the folded or unfolded state (Fig. 2A) and that the unfolded- (*Q*_u_) and folded-state minima (*Q*_f_) are separated by a transition-state maximum (Q*), whose locations are indicated by the dashed lines (Fig. 2B). These results represent a typical two-state behavior in the sense of the original, macrostate cooperativity concept.

The transition path is a portion of the trajectory that starts from an unfolded configuration (*Q*(***r***) < *Q*_u_) and ends at a folded one (*Q*(***r***) > *Q*_f_) without recrossing the *Q* = *Q*_u_ line. To detect cooperativity among multiple amino acids, we introduce a time-dependent correlation,1
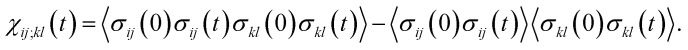
Here, the time *t* is measured relative to the beginning of the transition path (*i.e.*, *Q*(***r***(*t*)) = *Q*_u_ at *t* = 0); *σ*_*ij*_(*t*) is equal to 1 when there is a contact between a pair of amino acids *i* and *j* at time *t*, and equal to −1 otherwise; *σ*_*ij*_(0)*σ*_*ij*_(*t*) therefore varies from 1 to −1 when a contact absent at time *t* = 0 is formed at time *t*; and the angular brackets denote an average over the configurations at *t* = 0. By definition, *χ*_*ij*;*kl*_(*t*) = 0 when the contact formations of (*i*, *j*) and (*k*, *l*) amino-acid pairs occur independently. Therefore, *χ*_*ij*;*kl*_(*t*) > 0 indicates the existence of positive cooperativity between (*i*, *j*) and (*k*, *l*) amino-acid pairs at time *t*. We also introduce
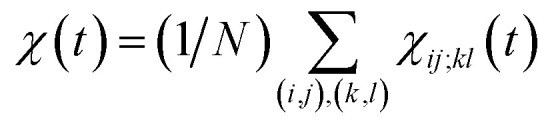
 averaged over all the pairs forming native amino-acid contacts, with *N* denoting the number of those pairs, which is a measure of an overall strength of the cooperativity present in a protein at time *t*. The time-dependent correlation *χ*(*t*), when viewed as a multipoint time-correlation function, is an analog of the dynamic susceptibility used for probing cooperative dynamics in glass-forming supercooled systems.^22–24^

We computed *χ*(*t*) for the transition path (cyan curve in Fig. 2C) by averaging over all the transition paths identified in each system. We also computed *χ*(*t*) for the unfolded state (magenta curve in Fig. 2C) using the trajectory parts that are close to *Q* = *Q*_u_ (painted magenta in Fig. 2B). We find that, while *χ*(*t*) for the unfolded state remains small at all the times, the one for the transition path develops a significant peak. We confirmed that the peak indeed originates from the correlation of distinct amino-acid pairs by comparing the diagonal ((*i*, *j*) = (*k*, *l*)) and off-diagonal ((*i*, *j*) ≠ (*k*, *l*)) contributions to *χ*(*t*) (Fig. S9†), to be denoted as *χ*_diag_(*t*) and *χ*_off-diag_(*t*) in the following. Thus, the growth of the amino-acid correlation is a distinguishing characteristic unique to the transition path. Such a behavior of *χ*(*t*) as a function of time closely resembles that of a microscopic measure of “thermodynamic cooperativity” *versus* temperature,^25^ and the cooperativity described by *χ*(*t*) may be termed the dynamic cooperativity. Our observation is also consistent with the recent NMR measurements demonstrating that the amino acids forming key contacts in the transition state interact not simultaneously in the denatured state.^26^

**Fig. 3:**
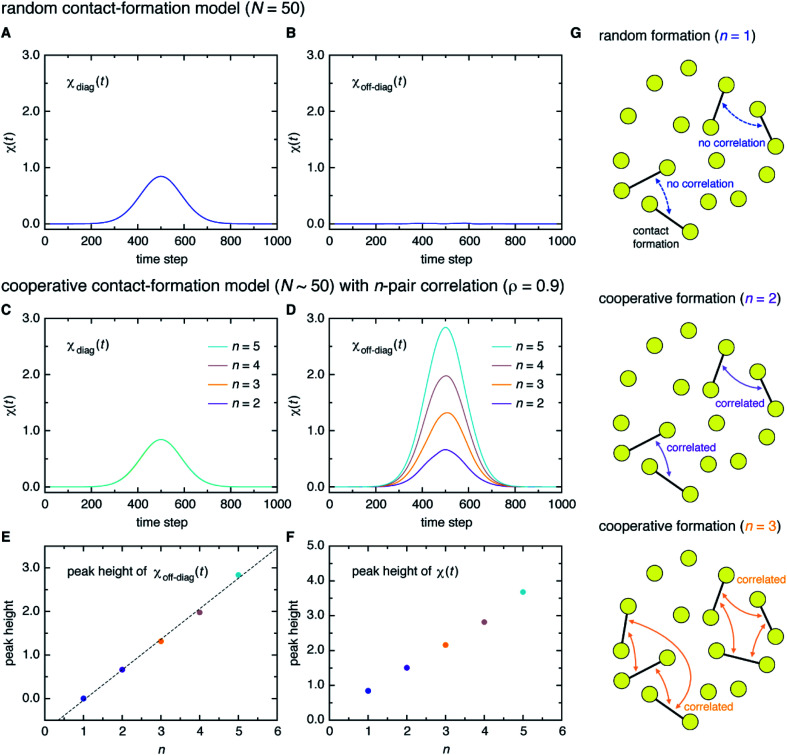
Random *versus* cooperative contact-formation models. (A and B) The self-component *χ*_diag_(*t*) (A) and the distinct component *χ*_off-diag_(*t*) (B) computed from the random contact-formation model of *N* = 50. (C and D) Corresponding results from the cooperative contact-formation model that incorporates correlation (*ρ* = 0.9) between *n* amino-acid pairs. (E and F) The peak height of *χ*_off-diag_(*t*) along with a linear fit denoted by the dashed line (E), and the peak height of *χ*(*t*) (*F*) for *n* = 1 to 5 (*n* = 1 corresponds to the random contact-formation model). (G) Schematic illustration of the random (*n* = 1) and cooperative (*n* = 2 and 3) contact formation in which vertices (yellow circles) represent amino acids and edges (black lines) represent the formation of native amino-acid contacts.

Here, a digression might be useful to better understand the nature of *χ*(*t*) = *χ*_diag_(*t*) + *χ*_off-diag_(*t*) since a peak in *χ*(*t*) may arise from a trivial reason, *i.e.*, just from the fact that a number of amino-acid contacts are formed roughly at the same time (in fact, the folding occurs within quite a short duration of time as can be inferred from Fig. 2A). We introduce a simple random model in which amino-acid pair contact formations are assumed to occur at random, Gaussian distributed times about the middle of the transition path. We find that *χ*(*t*) of this model exhibits a peak whose height is about 1. However, since this model does not incorporate any correlations between distinct amino-acid pairs, such a peak entirely reflects the “self” term, *i.e.*, *χ*(*t*) ≈ *χ*_diag_(*t*) ≈ 1 and *χ*_off-diag_(*t*) ≈ 0 (Fig. 3A and B). Thus, the mere presence of a peak in *χ*(*t*) does not warrant the existence of cooperative processes. We next consider an extended model in which correlations (characterized by the correlation coefficient *ρ*) are imposed between contact formation times of *n* amino-acid pairs. This model can be implemented by using the *n*-variate Gaussian distribution.^9^ (We notice that *n* = 1 corresponds to the random model.) We find for the model of *ρ* = 0.9 that, whereas *χ*_diag_(*t*) remains the same as that of the random model, the peak of *χ*_off-diag_(*t*) increases linearly with *n* and that the peak height of *χ*(*t*) provides a very rough estimate of the average number of correlated contact pairs (Fig. 3C to F). Thus, *χ*(*t*) conforming to *χ*_off-diag_(*t*) ≫ 1, which holds in the protein systems studied here (Fig. S9†), indeed indicates the presence of highly cooperative amino-acid contact formation.

**Fig. 4:**
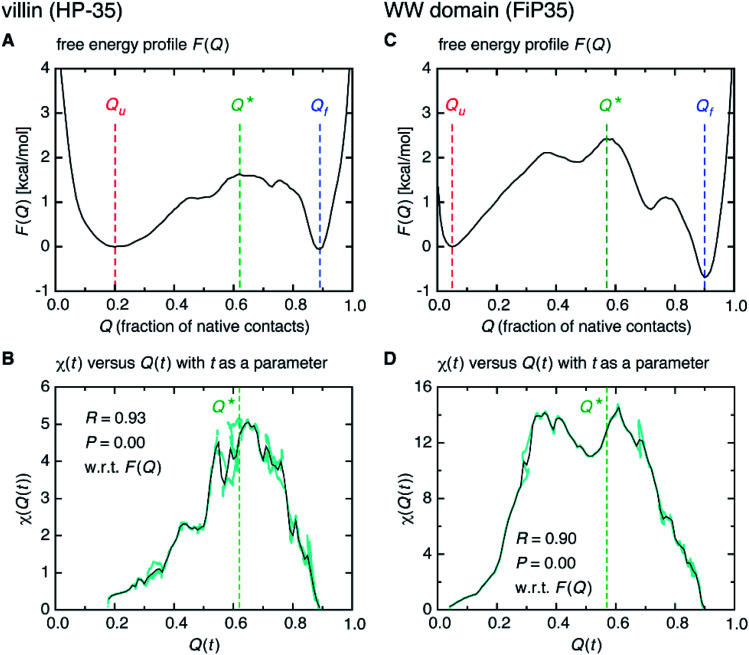
Connection between the macrostate (thermodynamic) and microscopic (dynamic) cooperativity. (A) Folding free energy profile *F*(*Q*) *versus Q* of the villin headpiece subdomain (HP-35). (B) Parametric plot of *χ*(*t*) *versus Q*(*t*) with *t* as a parameter (cyan filled circles). The black solid line was obtained after taking the average along the vertical direction for each *Q* = *Q*(*t*). (C and D) Corresponding results for the WW domain (FiP35).

Interestingly, we find that the time at which the amino-acid cooperativity attains its maximum size corresponds to the time when the system crosses the transition state. Not only can this be identified in Fig. 2C, but it can be also observed in the corresponding figures for the other systems, in which the average time t* the transition state is reached at (*i.e.*, *Q*(t*) = Q* with *Q*(*t*) = 〈*Q*(***r***(*t*))〉) is indicated by the vertical dashed line. This implies that the transition state can be characterized as the state in which the amino-acid cooperativity is maximal. To further corroborate this observation, we plotted *χ*(*t*) as a function of *Q*(*t*) with *t* as a parameter. The resulting *χ*(*Q*(*t*)) profile is shown and compared with the free energy profile *F*(*Q*) in Fig. 4A and B. We find that *χ*(*Q*(*t*)) closely traces *F*(*Q*) not only in the transition-state region (*Q* = Q*), but also in the whole *Q* range (*Q*_u_ ≤ *Q* ≤ *Q*_f_) it is defined (Pearson's correlation coefficient is *R* = 0.93; corresponding results for the other systems are shown in Fig. 4C, D and in Fig. S10†). This is a nontrivial result since *χ*(*Q*(*t*)) is purely a dynamic quantity, and provides evidence demonstrating that the macrostate, thermodynamic cooperativity (brought about by the presence of the transition-state barrier) is connected to the microscopic, dynamic cooperativity (characterized by *χ*(*t*)).

**Fig. 5:**
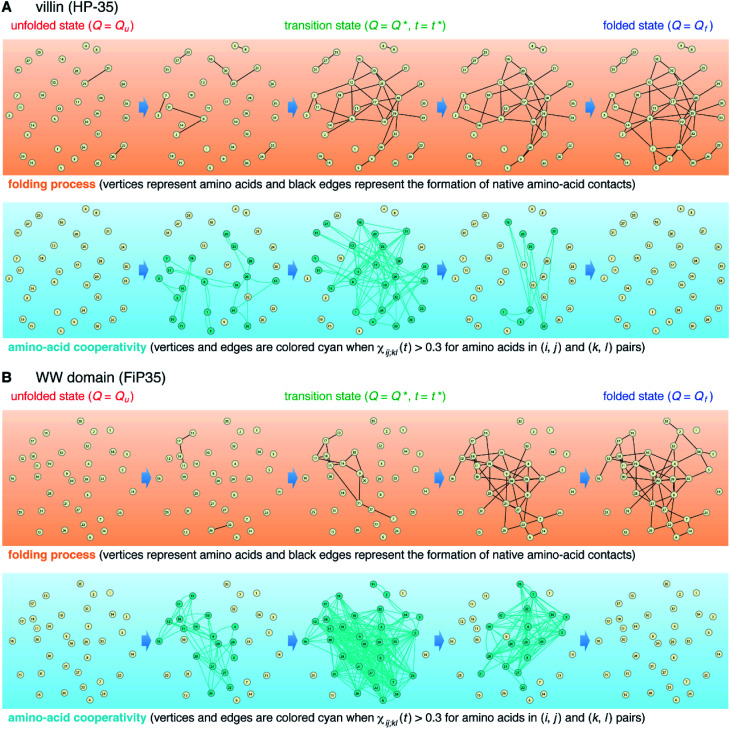
Network representation of the folding transition path. (A) Upper section: network representation of the folding process of the villin headpiece subdomain (HP-35) in which vertices (yellow circles) represent amino acids and edges (black lines) represent the formation of native amino-acid contacts. Lower section: network representation of the time-dependent amino-acid cooperativity in which vertices and edges are colored cyan when *χ*_*ij*;*kl*_(*t*) > 0.3 for amino acids in (*i*, *j*) and (*k*, *l*) pairs. (B) Corresponding results for the WW domain (FiP35).

The element-wise correlation *χ*_*ij*;*kl*_(*t*) at *t* = t* (Fig. 2D) quantifies the strength of communication between individual amino-acid pairs. To facilitate its visual understanding, we present in Fig. 5A network representations of protein configurations during the transition path. In the upper section, the vertices (yellow circles) refer to amino acids and the edges (black lines) represent the formation of native amino-acid contacts; the folding process implies an increase in the number of black edges. In the lower section, the vertices and edges are colored cyan when *χ*_*ij*;*kl*_(*t*) > 0.3 for those amino acids in (*i*, *j*) and (*k*, *l*) pairs (this criterion was chosen since such large amino-acid correlation is barely observed in the unfolded state, as shown in Fig. S11†). The growth of the amino-acid correlation toward the transition state and its subsequent diminution are clearly visible in the network graphs.

Further insights into the amino-acid cooperativity, which are smeared in *χ*(*t*) after summing over all the pairs, can be gained through the analysis of individual *χ*_*ij*;*kl*_(*t*) elements. For example, *χ*(*t*) can be decomposed into the main-chain and side-chain contributions by examining which of the main-chain and side-chain contacts is mainly involved in the (*i*, *j*) and (*k*, *l*) amino-acid pairs, and we find that the magnitude of those contributions is comparable (Fig. S12†). The peak time 
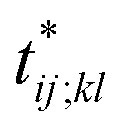
 for each *χ*_*ij*;*kl*_(*t*) element can also be introduced. We observe that 
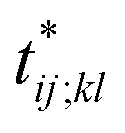
 values are dispersed around the average peak time t* (Fig. S13†). Again, this is a dynamical analog of the thermodynamic transition in which residue-dependent variations were identified in the transition midpoint temperature.^27^

## Discussion

The fact that the folding transition state can be characterized as the state of maximum cooperativity is, to the best of our knowledge, a novel view. However, it is in fact quite natural once the existence of such cooperativity is cognized. This is because the protein configurations exhibiting the maximum internal correlations will be the ones with the lowest probability of forming spontaneously. This new view in turn implies that the transition state barrier height should be an increasing function of the strength of the cooperativity. This is indeed the case as demonstrated in Fig. 4, which connects the microscopic cooperativity (characterized by *χ*(*t*)) and the macrostate two-state folding cooperativity (brought about by the presence of the transition-state barrier in *F*(*Q*)).

Our current view of protein folding owes much to the funneled energy landscape perspective.^28–30^ This perspective asserts that, in order to resolve Levinthal's paradox,^31,32^ folding should not be a random conformation search; it must be energetically biased. However, the landscape perspective does not provide a clear picture of the transition-state barrier responsible for the emergence of cooperative two-state folding: the barrier is ascribed as being due to a “mismatch” between the energy gain and the entropy loss at the middle of the funneled landscape.^33^ As we argued here, the folding transition state comes out naturally as the state of the maximum microscopic cooperativity by realizing that the amino acid contact formation is not a random process, but occurs on a multiple-interaction basis. In this sense, the new view for the folding transition state represents an extension of the landscape perspective.

While native contacts are of primary interest in protein folding studies, non-native contacts can in principle contribute to the time-dependent amino-acid cooperativity discussed in the present work. This is because *χ*_*ij*;*kl*_(*t*) defined in eqn (1) is invariant under the sign change, *σ*_*ij*_(*t*) → −*σ*_*ij*_(*t*): *σ*_*ij*_(0)*σ*_*ij*_(*t*) varies from 1 to −1 not only when a contact absent at time *t* = 0 (*σ*_*ij*_(0) = −1) is formed at time *t* (*σ*_*ij*_(*t*) = 1), but also when a contact present at time *t* = 0 (*σ*_*ij*_(0) = 1) is broken at time *t* (*σ*_*ij*_(*t*) = −1). Therefore, if there exist a number of non-native contacts that are highly populated in the unfolded state but are broken during the folding process, they would contribute to *χ*(*t*). For the systems studied here, the existence of highly populated non-native contacts was not detected, and we cannot illustrate such a possibility. Nevertheless, it is important to realize that the amino-acid cooperativity does not necessary refer to the formation of contacts; the breaking of contacts can also occur cooperatively.

Finally, we present a possible experimental method for the detection of the cooperative contact formation of multiple amino acids by using a kind of Kirkwood relation that connects fluctuations and response. For this purpose, we introduce a two-point time correlation function *F*(*t*) = 〈*q*(*t*)〉 with 
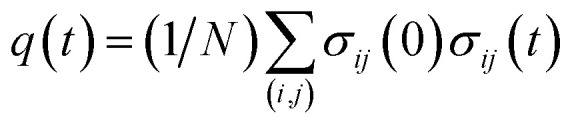
. This function describes how on average the native contacts are being formed as the folding proceeds. The multipoint function *χ*(*t*) capturing the time-dependent cooperativity can be written as the fluctuations around the average folding dynamics: *χ*(*t*) = *N*〈*δq*(*t*)^2^〉 in which *δq*(*t*) = *q*(*t*) − 〈*q*(*t*)〉. Let us introduce a susceptibility defined as the response of *F*(*t*) to a perturbation *φ* (such as a change in temperature): *χ*_*φ*_(*t*) = ∂*F*(*t*)/∂*φ*. It was demonstrated for dielectric and density fluctuations in glass-forming systems that *χ*_*φ*_(*t*)^2^ exhibits essentially the same dynamics as *χ*(*t*).^34^ Since the average function *F*(*t*) is intimately related to the “shape” function of the transition path that is now experimentally accessible,^35^ measuring *χ*_*φ*_(*t*) by varying experimental conditions will provide experimental evidence of the microscopic cooperativity in protein folding.

## Conclusions

Cooperativity in complex systems is typically described at a macrostate level, and its characterization in molecular terms has been very challenging. In the present work, we succeed in identifying time-dependent cooperativity among multiple amino acids concealed in the folding transition path, and argue how it might be connected to the macrostate cooperative behavior. The use of the multipoint correlation functions is essential in this regard, since a cooperative nature of fluctuating processes occurring at two distinct sites cannot be disclosed by conventional, two-point correlation functions. Since cooperativity pervades complex biological phenomena—the most notable example being allostery^36^—the multipoint correlation function approach will bring out novel microscopic insights into those complex processes.

## Author contributions

S.-H. C. and S. H. designed the research, conducted the research, and wrote the manuscript.

## Conflicts of interest

There are no conflicts to declare.

## Supplementary Material

SC-012-D0SC07025D-s001
