# Supplementary material for: Time-dependent communication between multiple amino acids during protein folding
Source: Chem Sci. 2021 Mar 24;12(16):5944–51. doi: 10.1039/d0sc07025d (PMC8871807; doi:10.1039/d0sc07025d)
Supplement: SC-012-D0SC07025D-s001 [file SC-012-D0SC07025D-s001.pdf]

Electronic Supplementary Material (ESI) for Chemical Science.
This journal is © The Royal Society of Chemistry 2021

Electronic Supplementary Information for

# Time-Dependent Communication between Multiple Amino Acids during Protein Folding

Song-Ho Chong and Sihyun Ham*

*Department of Chemistry, The Research Institute of Natural Sciences,*
*Sookmyung Women's University,*
*Cheongpa-ro 47-gil 100, Yongsan-ku, Seoul 04310, Korea*

Corresponding author:

*Sihyun Ham

**Address** Department of Chemistry
The Research Institute of Natural Sciences
Sookmyung Women's University
Cheongpa-ro 47-gil 100, Yongsan-ku, Seoul 04310, Korea

**Email** sihyun@sookmyung.ac.kr

**Phone** +82-2-710-9410

## Materials and Methods

**Materials.** We investigated the protein systems studied by Shaw and coworkers which have at least ~30 amino acid residues and exhibit a well-defiled transition state barrier. Figure 1 in the main text displays the 10 systems satisfying the criteria. The number of amino acid residues, the simulation temperature, and the aggregated simulation time for each system are presented in Table S1. The force fields used were FF99SB*-ILDN[S1-S3] for the villin and its mutant, FF99SB-ILDN[S1,S2] for the WW domain, and CHARMM22*[S4-S6] for the other systems; and the TIP3P water model[S7] was adopted for all the systems. Protein configurations saved with a $\Delta t$ = 0.2 ns time interval ($\Delta t$ = 1 ns for ubiquitin) were subjected to the analyses. This means, e.g., for the villin of ~400 μs simulation length, ~2 × $10^6$ configurations were analyzed.

**Fraction of native contacts.** We computed the fraction of native contacts $Q(\boldsymbol{r})$ for each protein configuration $\boldsymbol{r}$ following the procedure of ref. S8. We first constructed a list of native atom–atom contact pairs found in the native structure: a pair of heavy atoms $a$ and $b$ belonging to amino acid residues $i$ and $j$ is in a native contact if the distance between $a$ and $b$ is <4.5 Å and $|i-j| > 3$. Then, $Q(\boldsymbol{r})$ is given by

$$Q(\boldsymbol{r}) = \frac{1}{N_{\mathrm{atom-pair}}} \sum_{(a,b)} Q_{ab}(\boldsymbol{r})$$

with

$$Q_{ab}(\boldsymbol{r}) = \frac{1}{1 + \exp[\beta(r_{ab}(\boldsymbol{r}) - \lambda r_{ab}^{0})]}$$

Here, $N_{\mathrm{atom\text{-}pair}}$ is the total number of native atom–atom contact pairs; $r_{ab}(\boldsymbol{r})$ is the distance between $a$ and $b$ in a specific protein configuration $\boldsymbol{r}$; $r_{ab}^{0}$ is the distance between $a$ and $b$ in the native structure; and $\beta$ = 5 Å$^{-1}$ and $\lambda$ = 1.8. The native structure for each system, displayed in Fig. 1, was taken from the corresponding experimental structure in the Protein Data Bank, whose ID is listed in Table S1.

**Multipoint correlation function.** The variable $\sigma_{ij}$ introduced in defining the multipoint time-correlation function is more precisely given by the following expression based on the fraction of native atom–atom contacts $Q_{ab}(\boldsymbol{r})$ defined above:

$$\sigma_{ij}(\boldsymbol{r}) = 2Q_{ij}(\boldsymbol{r}) - 1$$

with

$$Q_{ij}(\boldsymbol{r}) = \frac{1}{N_{ij}} \sum_{(a,b)\in(i,j)} Q_{ab}(\boldsymbol{r})$$

Here, $a$ and $b$ refer to atoms forming a native contact, $i$ and $j$ denote amino acids to which they belong, and $N_{ij}$ is the number of native atom–atom contacts between amino acids $i$ and $j$. When all the native atom–atom contacts are formed between amino acids $i$ and $j$, $Q_{ij} = 1$ and hence $\sigma_{ij} = 1$; When none of them are present, on the other hand, $Q_{ij} = 0$ and hence $\sigma_{ij} = -1$. However, $Q_{ij}$ and $\sigma_{ij}$ can take fractional values when the native atom–atom contacts between amino acids $i$ and $j$ are only partially formed.

**Main-chain and side-chain contributions.** The decomposition of $\chi(t)$ into the main-chain and side-chain contributions was done as follows:

$$\chi(t) = \chi^{\text{main}}(t) + \chi^{\text{side}}(t)$$

with

$$\chi^{\text{main(side)}}(t) = \frac{1}{N} \sum_{(i,j),(k,l)} w_{ij;kl}^{\text{main(side)}} \chi_{ij;kl}(t)$$

Here, $w_{ij;kl}^{\text{main}}$ and $w_{ij;kl}^{\text{side}}$, satisfying $w_{ij;kl}^{\text{main}} + w_{ij;kl}^{\text{side}} = 1$, are the weights determined by the characters of the $(i, j)$ and $(k, l)$ amino acid contacts, which in turn are judged from the minimum heavy-atom distances. For example, if the contact between amino acids $i$ and $j$ is a main-chain/side-chain contact, it is characterized as the "MS" type; if the contact between amino acids $k$ and $l$ is a side-chain/side-chain contact, it is characterized as the "SS" type. Then, $w_{ij;kl}^{\text{main}}$ ($w_{ij;kl}^{\text{side}}$) is set to the total number of "M" ("S") divided by 4: $w_{ij;kl}^{\text{main}} = 1/4$ and $w_{ij;kl}^{\text{side}} = 3/4$ in the above example. The results shown in Fig. S12 were obtained in this manner.

**Random contact-formation model and its extension.** The random contact-formation model introduced in the main text is a mathematical model in which the formations of $N$ native contact pairs are assumed to occur at random, Gaussian distributed times during the transition path. The following is the self-explanatory pseudocode of the model:

```
ntrajectories=100       /* number of trajectory portions used */
ncontacts=50            /* number of native contact pairs considered */
nsteps=1000             /* length of the trajectory portions */

sigt=(0.5*nsteps)/5.0   /* width of Gaussian distribution of contact formation times */
sigf=(0.5*nsteps)/20.0  /* width of transition to contact for each pair */

sig=array[ncontacts,nsteps,ntrajectories]       /* corresponds to sigma in manuscript */
chi=array[ncontacts,ncontacts,nsteps]           /* corresponds to chi_ij;kl(t) in manuscript */

chit=array[nsteps]              /* corresponds to chi(t) in manuscript */
chit_diag=array[nsteps]         /* corresponds to chi_diag(t) in manuscript */
chit_off_diag=array[nsteps]     /* corresponds to chi_off_diag(t) in manuscript */

t=array[nsteps]

for i=0,nsteps-1:
  t[i]=i

for i=0,ntrajectories-1:
  for j=0,ncontacts-1:
    formationtime=nsteps/2+sigt*random_gauss()
    for k=0,nsteps-1:
      sig[j,k,i]=erf((t[k]-formationtime)/sigf) /* error function, ranges from -1 to +1 */

for j=0,ncontacts-1:
  for k=0,ncontacts-1:
    for ti=0,nsteps-1:
      c=0
      c1=0
      c2=0
      for i=0,ntrajectories-1:
        c=c+sig[j,0,i]*sig[j,ti,i]*sig[k,0,i]*sig[k,ti,i]
        c1=c1+sig[j,0,i]*sig[j,ti,i]
        c2=c2+sig[k,0,i]*sig[k,ti,i]
      chi[j,k,ti]=c/ntraj - (c1/ntraj)*(c2/ntraj)

for ti=0,nsteps-1:
  c1=0
  c2=0
  for i=0,ncontacts-1:
    for j=0,ncontacts-1:
      if i==j:
        c1=c1+chi[i,j,ti]
      else:
        c2=c2+chi[i,j,ti]
  chit_diag[ti]=c1/ncontacts
  chit_non_diag[ti]=c2/ncontacts
  chit[ti]=chit_diag[ti]+chit_non_diag[ti]
```

The results shown in Fig. 3A and B were obtained with $N = 50$, but we also analyzed $N = 100$ to confirm that results do not significantly depend on $N$. We also examined the number of trajectories ($n_{\mathrm{traj}}$) of 100, 1000 and 10000 to check the convergence.

This model is easily extendable to incorporate correlations between $n$ amino-acid pairs: By using the $n$-variate Gaussian distribution, we can impose correlations

(characterized by the correlation coefficient $\rho$) between contact formation times of $n$ pairs. This can be implemented, e.g., for the case of $n$ = 3, by replacing the part enclosed by the cyan square box in the above pseudocode with

```
rho = 0.9       /* correlation coefficient between contact formation times */

mean=array[3]
cov=array[3,3]

mean=[0.0,0.0,0.0]
cov=[[1.0,rho,rho],[rho,1.0,rho],[rho,rho,1.0]]

for i=0,ntrajectories-1:
  for j=0,ncontacts/3-1:
    formationtime1,formationtime2,formationtime3=nsteps/2+sigt*random_gauss_multivariate(mean,cov)
    for k=0,nsteps-1:
      sig[3*j+0,k,i]=erf((t[k]-formationtime1)/sigf)
      sig[3*j+1,k,i]=erf((t[k]-formationtime2)/sigf)
      sig[3*j+2,k,i]=erf((t[k]-formationtime3)/sigf)
```

The results shown in Fig. 3C to F were obtained from the extended model of $\rho$ = 0.9 with $N \sim 50$ up to $n$ = 5; we used $N$ = 50 for $n$ = 2 and 5, $N$ = 51 for $n$ = 3, and $N$ = 52 for $n$ = 4. We also note that the original random model corresponds to $n$ = 1.

**Network representation.** The network representations in Fig. 5 and in Figs. S1 to S8 were generated using the Python library graph tool (https://graph-tool.skewed.de).

## References


S1. V. Hornak et al., *Proteins*, 2006, **65**, 712–725.

S2. K. Lindorff-Larsen et al., *Proteins*, 2010, **78**, 1950–1958.

S3. R. B. Best and G. Hummer, *J. Phys. Chem. B*, 2009, **113**, 9004–9015.

S4. A. D. MacKerell Jr. et al., *J. Phys. Chem. B*, 1998, **102**, 3586–3616.

S5. A. D. MacKerell Jr., M. Feig and C. L. Brooks III, *J. Comput. Chem.*, 2004, **25**, 1400–1415.

S6. S. Piana, K. Lindorff-Larsen and D. E. Shaw, *Biophys. J.*, 2011, **100**, L47–L49.

S7. W. L. Jorgensen, J. Chandrasekhar, J. D. Madura, R. W. Impey and M. L. Klein, *J. Chem. Phys.*, 1983, **79**, 926–935.

S8. R. B. Best, G. Hummer and W. A. Eaton, *Proc. Natl. Acad. Sci. U.S.A.*, 2005, **102**, 7517–7522.

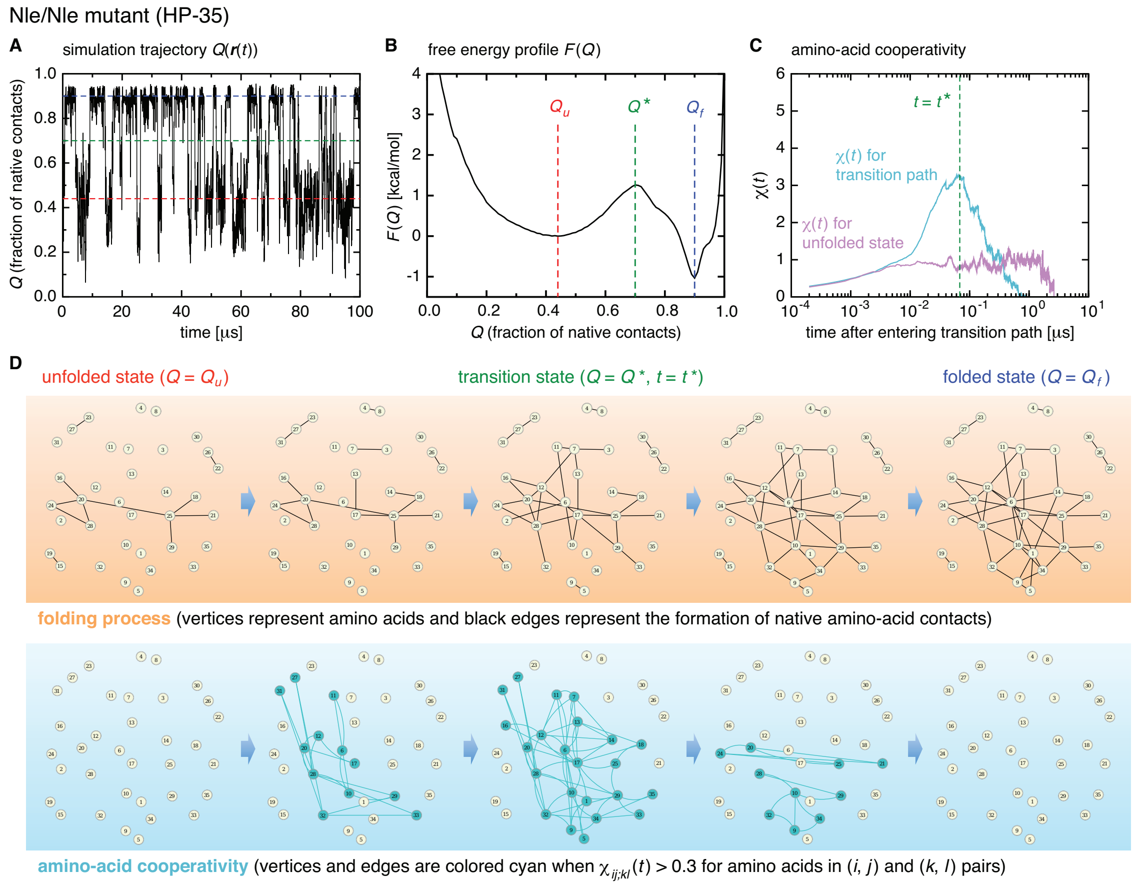


Figure S1: Time-dependent cooperativity between multiple amino acids in the mutant of villin (HP-35). (A) Fraction of the native amino-acid contacts $Q(\mathbf{r}(t))$ for the protein configuration $\mathbf{r}(t)$ at time $t$ for a 100 $\mu$s portion of the simulation trajectory. (B) Folding free energy profile $F(Q)$ versus $Q$. (C) $\chi(t)$ for the transition path (colored cyan) and for the unfolded state (colored magenta) on a logarithmic timescale. (D) Upper section: Network representation of the folding process in which vertices (yellow circles) represent amino acids and edges (black lines) represent the formation of native amino-acid contacts. Lower section: Network representation of the time-dependent amino-acid cooperativity in which vertices and edges are colored cyan when $\chi_{ij;kl}(t) > 0.3$ for amino acids in $(i, j)$ and $(k, l)$ pairs.

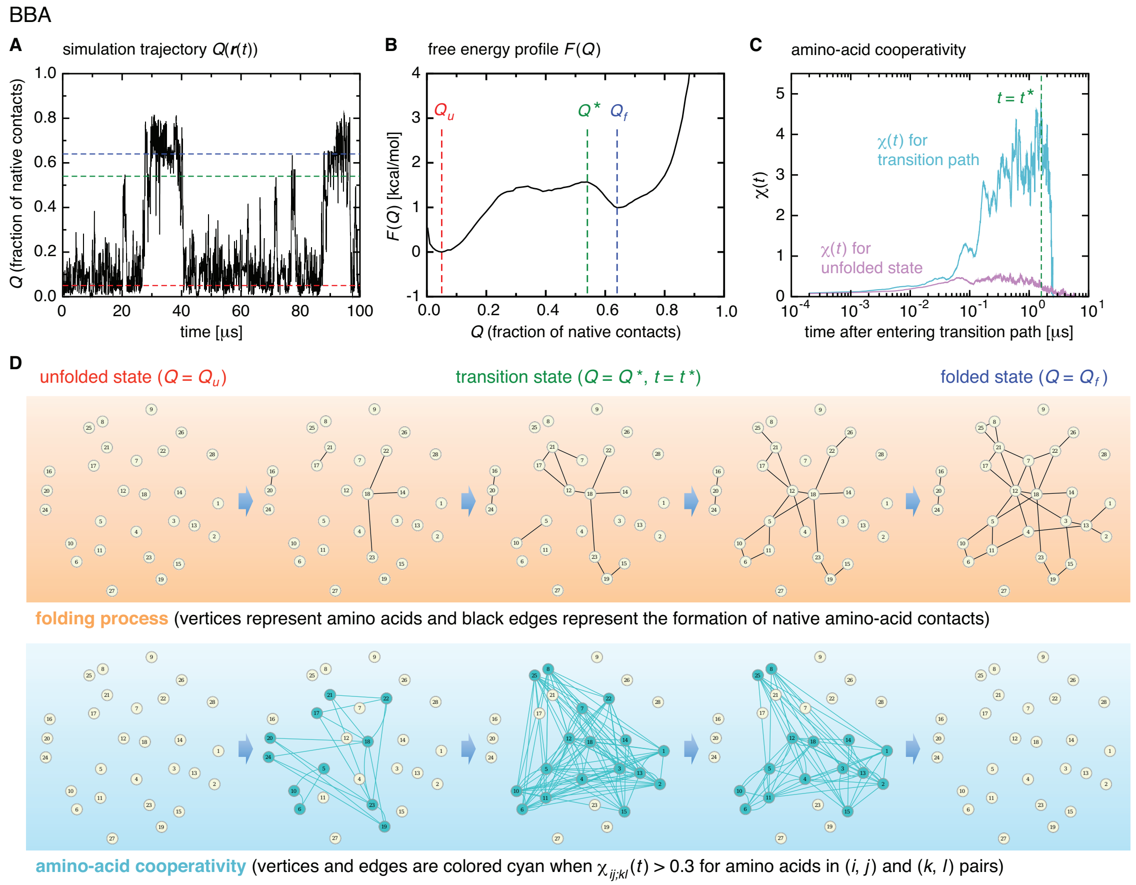


Figure S2: Time-dependent cooperativity between multiple amino acids in BBA. (A) Fraction of the native amino-acid contacts $Q(\mathbf{r}(t))$ for the protein configuration $\mathbf{r}(t)$ at time $t$ for a 100 $\mu$s portion of the simulation trajectory. (B) Folding free energy profile $F(Q)$ versus $Q$. (C) $\chi(t)$ for the transition path (colored cyan) and for the unfolded state (colored magenta) on a logarithmic timescale. (D) Upper section: Network representation of the folding process in which vertices (yellow circles) represent amino acids and edges (black lines) represent the formation of native amino-acid contacts. Lower section: Network representation of the time-dependent amino-acid cooperativity in which vertices and edges are colored cyan when $\chi_{ij;kl}(t) > 0.3$ for amino acids in $(i, j)$ and $(k, l)$ pairs.

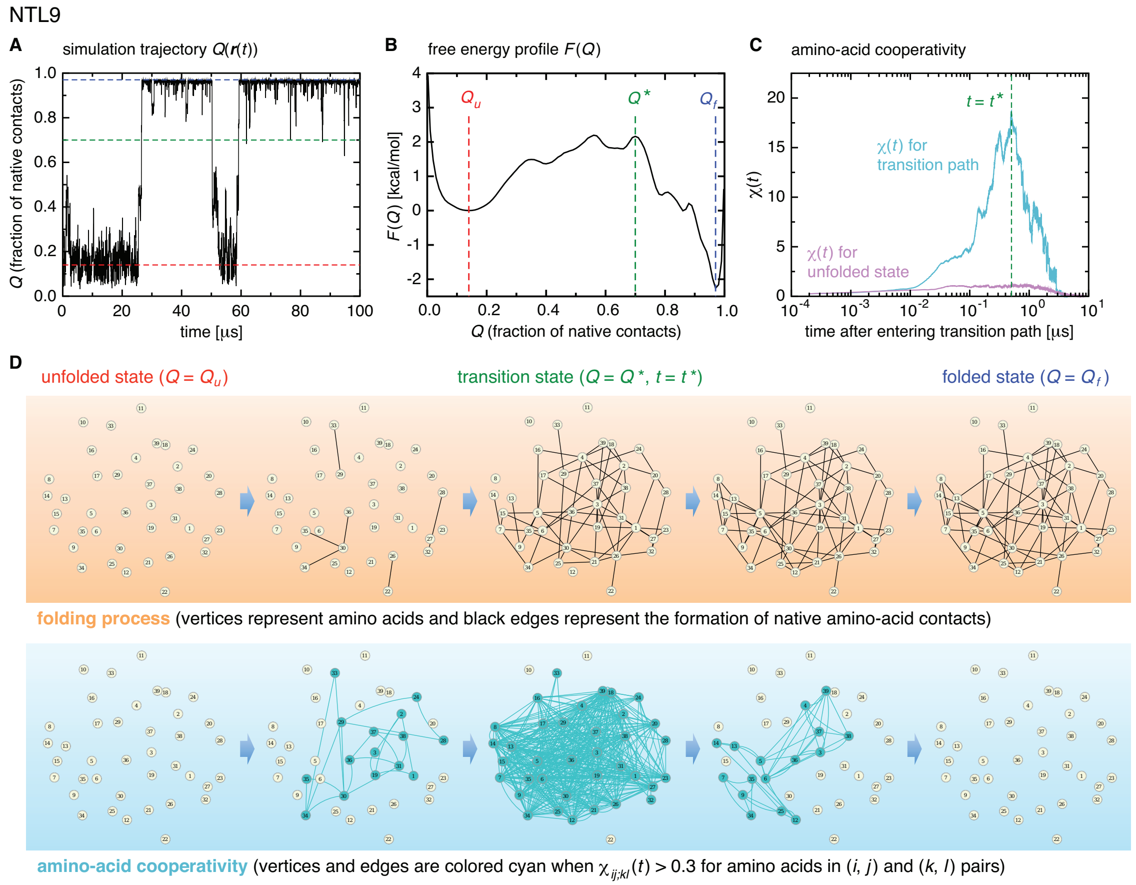


Figure S3: Time-dependent cooperativity between multiple amino acids in NTL9. (A) Fraction of the native amino-acid contacts $Q(\mathbf{r}(t))$ for the protein configuration $\mathbf{r}(t)$ at time $t$ for a 100 $\mu$s portion of the simulation trajectory. (B) Folding free energy profile $F(Q)$ versus $Q$. (C) $\chi(t)$ for the transition path (colored cyan) and for the unfolded state (colored magenta) on a logarithmic timescale. (D) Upper section: Network representation of the folding process in which vertices (yellow circles) represent amino acids and edges (black lines) represent the formation of native amino-acid contacts. Lower section: Network representation of the time-dependent amino-acid cooperativity in which vertices and edges are colored cyan when $\chi_{ij;kl}(t) > 0.3$ for amino acids in $(i, j)$ and $(k, l)$ pairs.

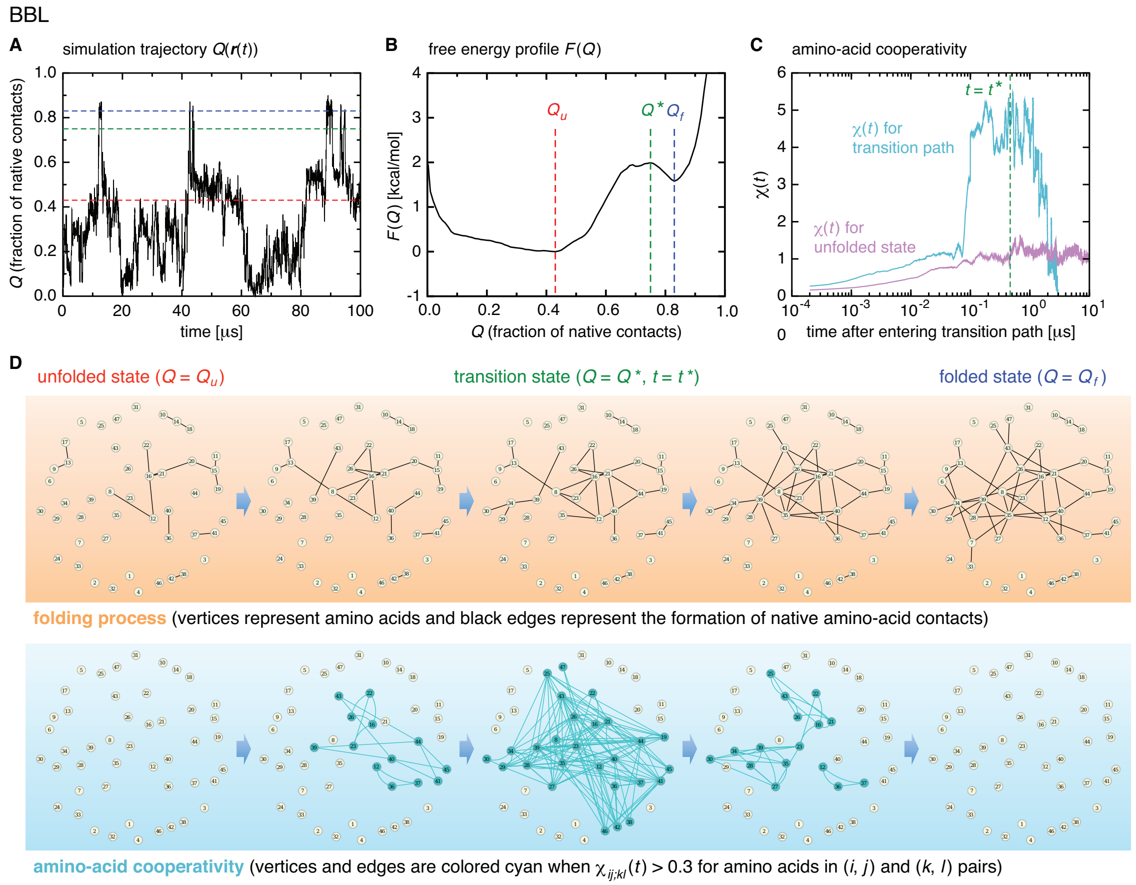


Figure S4: Time-dependent cooperativity between multiple amino acids in BBL. (A) Fraction of the native amino-acid contacts $Q(\mathbf{r}(t))$ for the protein configuration $\mathbf{r}(t)$ at time $t$ for a 100 $\mu$s portion of the simulation trajectory. (B) Folding free energy profile $F(Q)$ versus $Q$. (C) $\chi(t)$ for the transition path (colored cyan) and for the unfolded state (colored magenta) on a logarithmic timescale. (D) Upper section: Network representation of the folding process in which vertices (yellow circles) represent amino acids and edges (black lines) represent the formation of native amino-acid contacts. Lower section: Network representation of the time-dependent amino-acid cooperativity in which vertices and edges are colored cyan when $\chi_{ij;kl}(t) > 0.3$ for amino acids in $(i, j)$ and $(k, l)$ pairs.

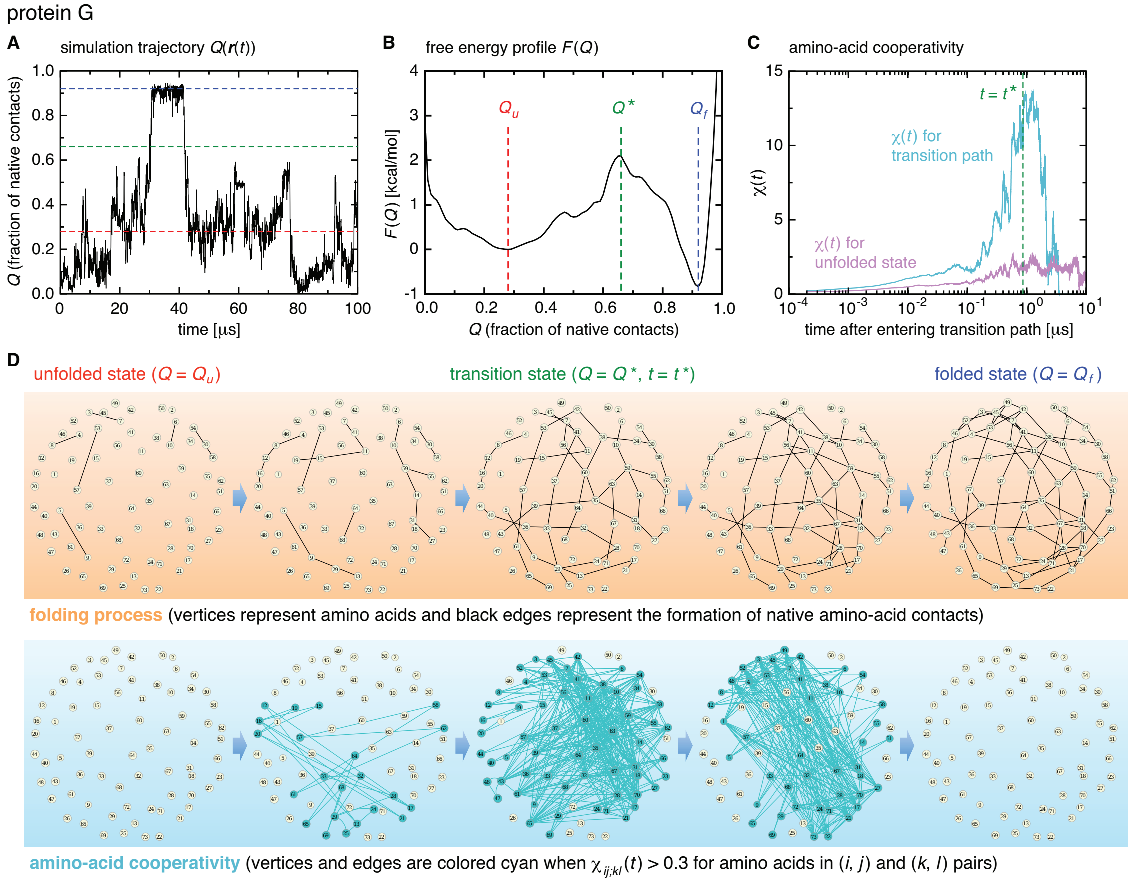


Figure S5: Time-dependent cooperativity between multiple amino acids in protein G. (A) Fraction of the native amino-acid contacts $Q(\mathbf{r}(t))$ for the protein configuration $\mathbf{r}(t)$ at time $t$ for a 100 $\mu$s portion of the simulation trajectory. (B) Folding free energy profile $F(Q)$ versus $Q$. (C) $\chi(t)$ for the transition path (colored cyan) and for the unfolded state (colored magenta) on a logarithmic timescale. (D) Upper section: Network representation of the folding process in which vertices (yellow circles) represent amino acids and edges (black lines) represent the formation of native amino-acid contacts. Lower section: Network representation of the time-dependent amino-acid cooperativity in which vertices and edges are colored cyan when $\chi_{ij;kl}(t) > 0.3$ for amino acids in $(i, j)$ and $(k, l)$ pairs.

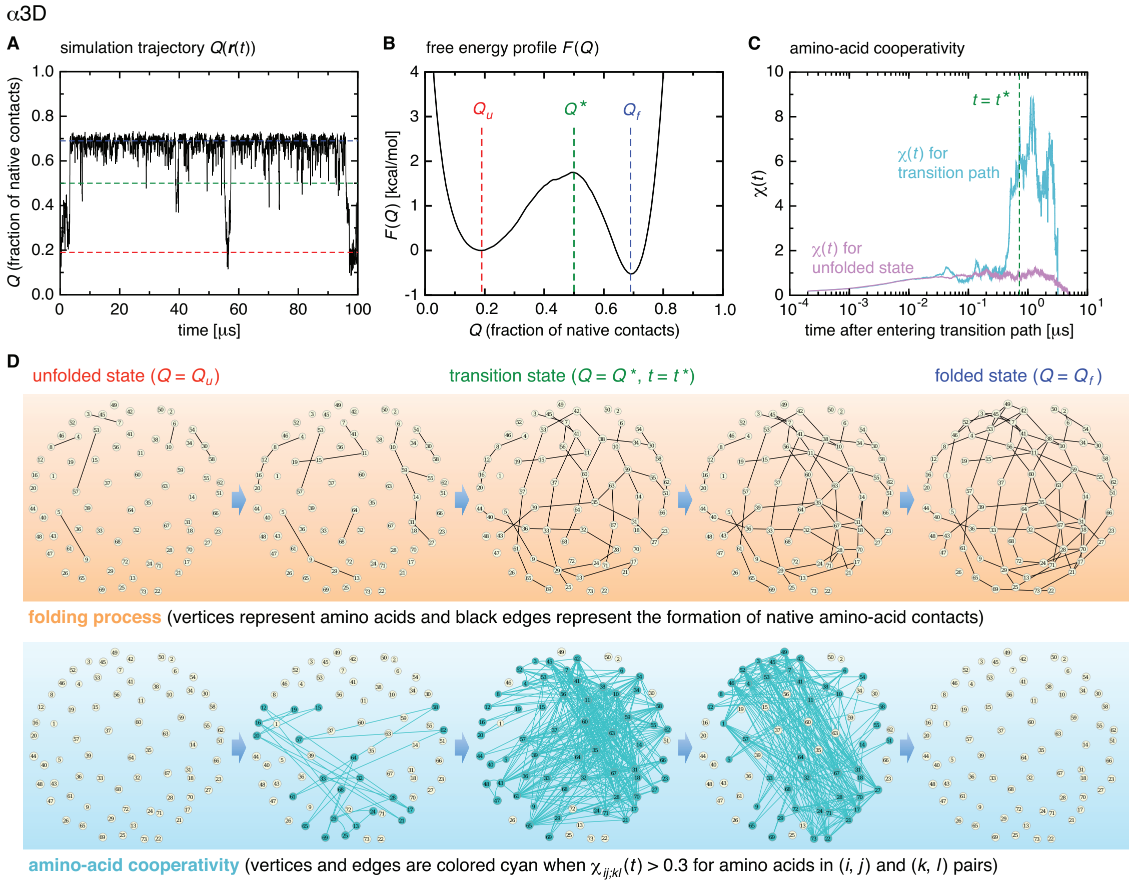


Figure S6: Time-dependent cooperativity between multiple amino acids in $\alpha$3D. (A) Fraction of the native amino-acid contacts $Q(\mathbf{r}(t))$ for the protein configuration $\mathbf{r}(t)$ at time $t$ for a 100 $\mu$s portion of the simulation trajectory. (B) Folding free energy profile $F(Q)$ versus $Q$. (C) $\chi(t)$ for the transition path (colored cyan) and for the unfolded state (colored magenta) on a logarithmic timescale. (D) Upper section: Network representation of the folding process in which vertices (yellow circles) represent amino acids and edges (black lines) represent the formation of native amino-acid contacts. Lower section: Network representation of the time-dependent amino-acid cooperativity in which vertices and edges are colored cyan when $\chi_{ij;kl}(t) > 0.3$ for amino acids in $(i, j)$ and $(k, l)$ pairs.

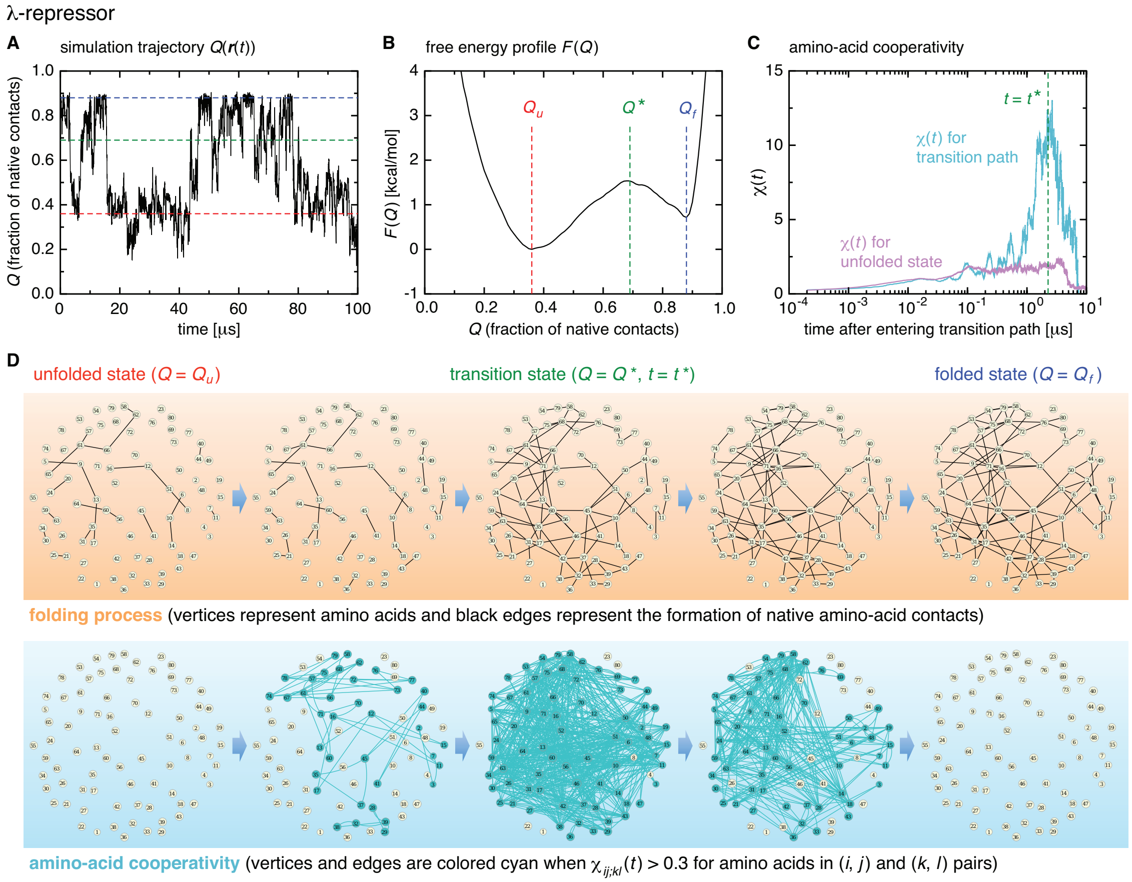


Figure S7: Time-dependent cooperativity between multiple amino acids in $\lambda$-repressor. (A) Fraction of the native amino-acid contacts $Q(\mathbf{r}(t))$ for the protein configuration $\mathbf{r}(t)$ at time $t$ for a 100 $\mu$s portion of the simulation trajectory. (B) Folding free energy profile $F(Q)$ versus $Q$. (C) $\chi(t)$ for the transition path (colored cyan) and for the unfolded state (colored magenta) on a logarithmic timescale. (D) Upper section: Network representation of the folding process in which vertices (yellow circles) represent amino acids and edges (black lines) represent the formation of native amino-acid contacts. Lower section: Network representation of the time-dependent amino-acid cooperativity in which vertices and edges are colored cyan when $\chi_{ij;kl}(t) > 0.3$ for amino acids in $(i, j)$ and $(k, l)$ pairs.

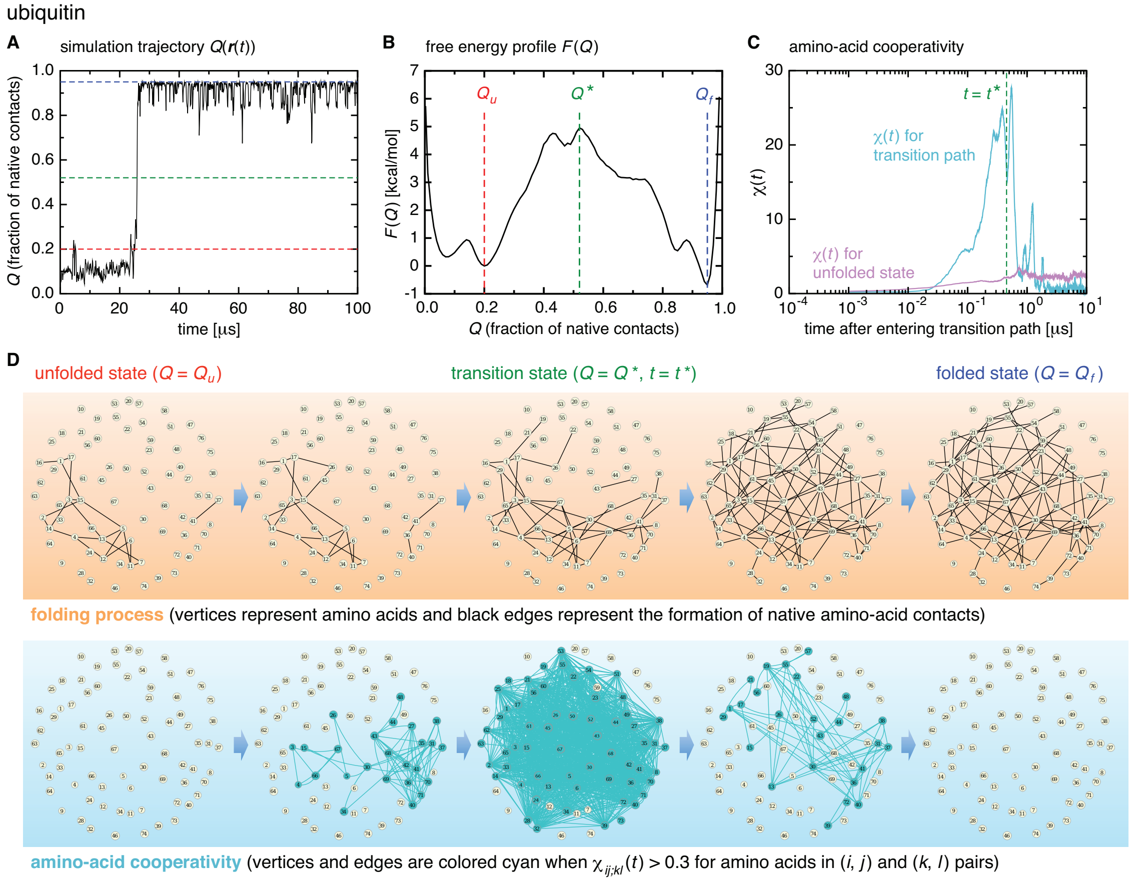


Figure S8: Time-dependent cooperativity between multiple amino acids in ubiquitin. (A) Fraction of the native amino-acid contacts $Q(\mathbf{r}(t))$ for the protein configuration $\mathbf{r}(t)$ at time $t$ for a 100 $\mu$s portion of the simulation trajectory. (B) Folding free energy profile $F(Q)$ versus $Q$. (C) $\chi(t)$ for the transition path (colored cyan) and for the unfolded state (colored magenta) on a logarithmic timescale. (D) Upper section: Network representation of the folding process in which vertices (yellow circles) represent amino acids and edges (black lines) represent the formation of native amino-acid contacts. Lower section: Network representation of the time-dependent amino-acid cooperativity in which vertices and edges are colored cyan when $\chi_{ij;kl}(t) > 0.3$ for amino acids in $(i, j)$ and $(k, l)$ pairs.

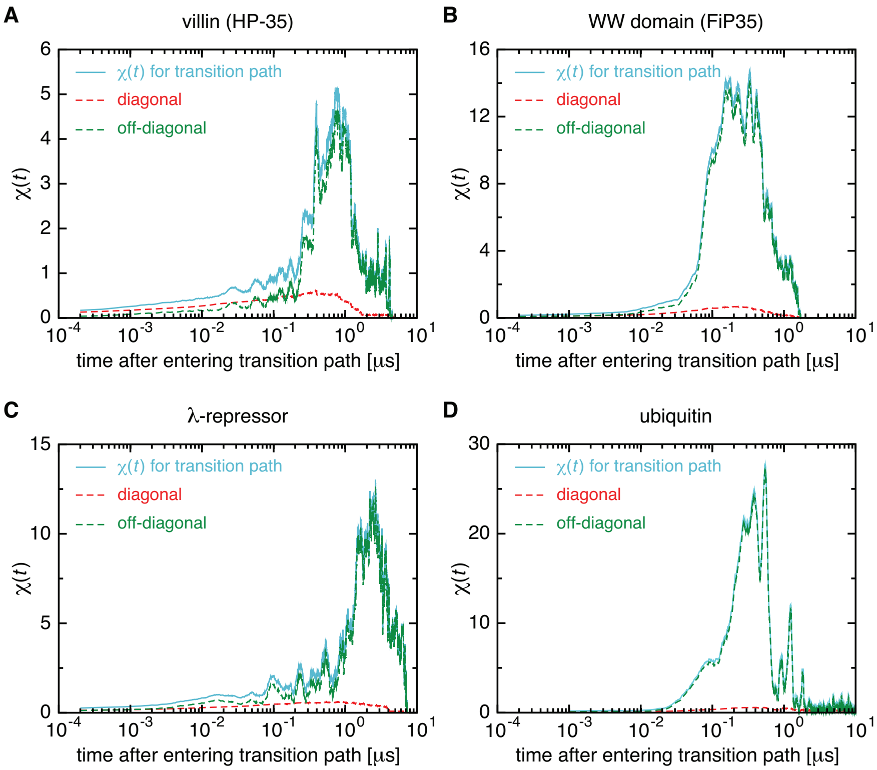


Figure S9: Diagonal versus off-diagonal element contributions to $\chi(t)$. (A) $\chi(t)$ for the transition path (colored cyan) of the villin headpiece subdomain is decomposed into the contributions from the diagonal elements ($\chi_{ij;kl}(t)$ with $(i,j) = (k,l)$; colored red) and from the off-diagonal elements ($\chi_{ij;kl}(t)$ with $(i,j) \neq (k,l)$; colored green). (B–D) Corresponding results for the WW domain (B), $\lambda$-repressor (C), and ubiquitin (D).

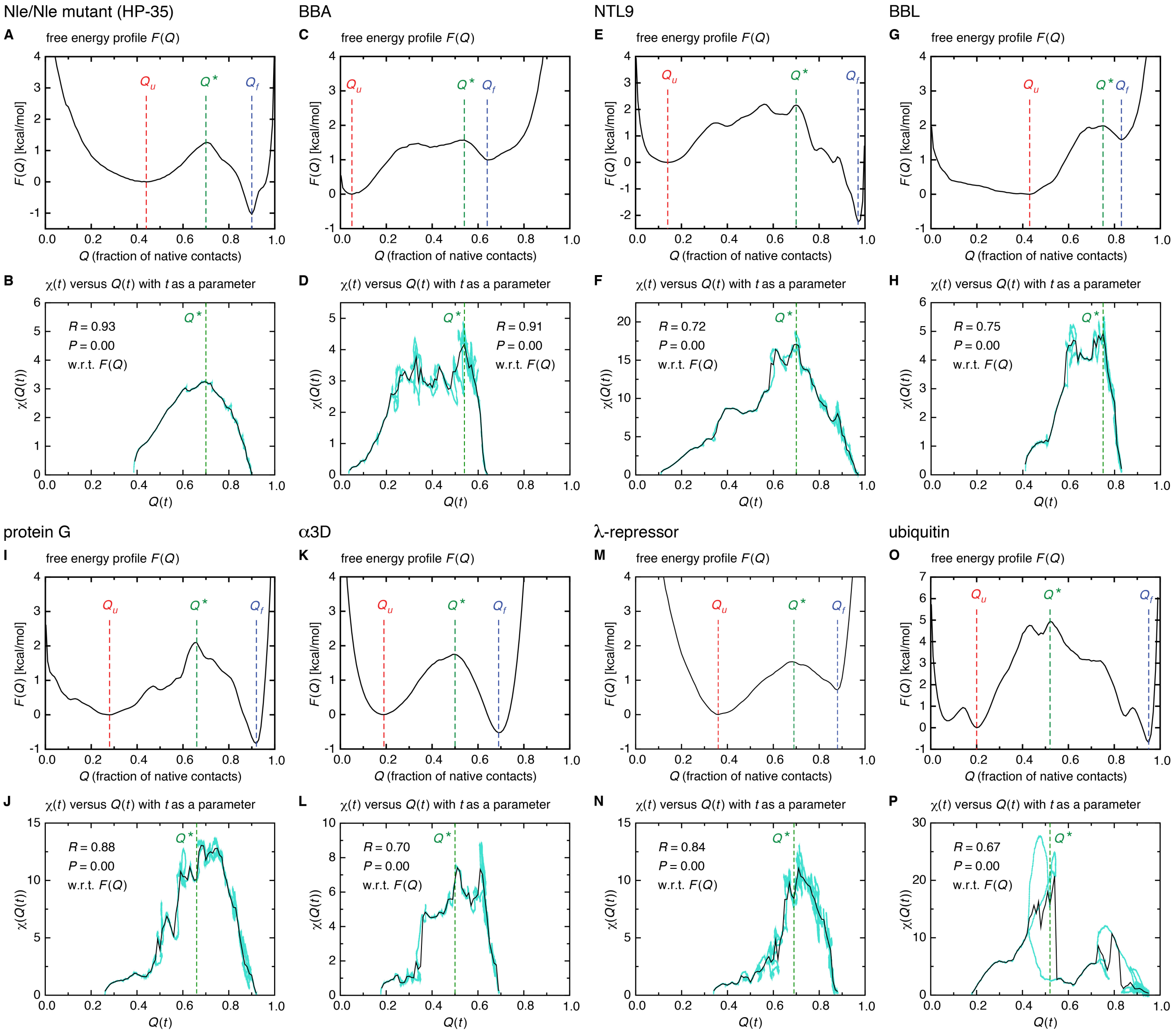


Figure S10: Connection between the macrostate (thermodynamic) and microscopic (dynamic) cooperativity. (A) Folding free energy profile $F(Q)$ versus $Q$ of the mutant of villin (HP-35). (B) Parametric plot of $\chi(t)$ versus $Q(t)$ with $t$ as a parameter (cyan filled circles). The black solid line was obtained after taking the average along the vertical direction for each $Q = Q(t)$. (C–P) Corresponding results for BBA (C,D), NTL9 (E,F), BBL (G,H), protein G (I,J), $\alpha$3D (K,L), $\lambda$-repressor (M,N) and ubiquitin (O,P).

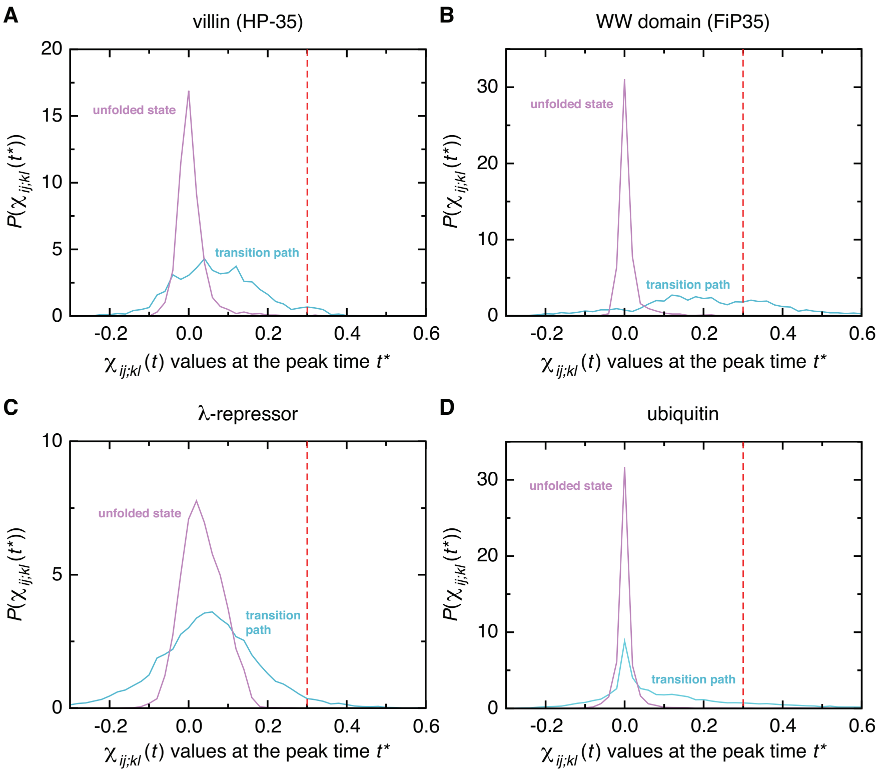


Figure S11: (A) Distribution of $\chi_{ij;kl}(t)$ values at the peak time $t^*$ of $\chi(t)$ for the transition path (colored cyan) and for the unfolded state (colored magenta) of the villin headpiece subdomain. The red vertical dashed line denotes the location of $\chi_{ij;kl}(t) = 0.3$ which is chosen as a criterion of the large amino-acid cooperativity. (B–D) Corresponding results for the WW domain (B), $\lambda$-repressor (C), and ubiquitin (D).

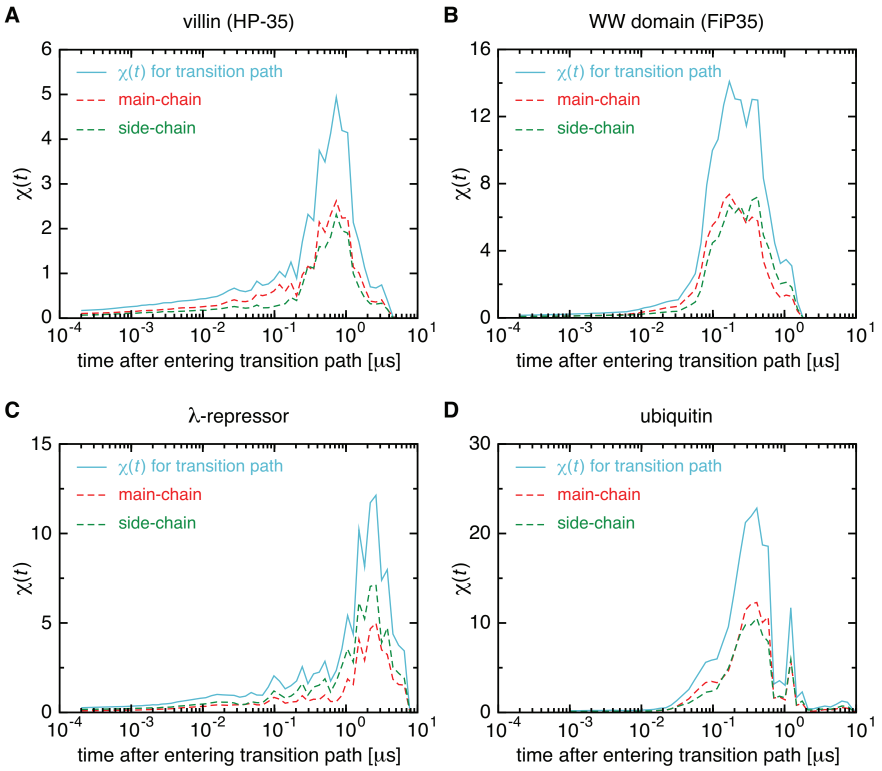


Figure S12: Main-chain versus side-chain contributions to $\chi(t)$. (A) $\chi(t)$ for the transition path (colored cyan) of the villin headpiece subdomain is decomposed into the main-chain (colored red) and side-chain (colored green) contributions. (B–D) Corresponding results for the WW domain (B), $\lambda$-repressor (C), and ubiquitin (D).

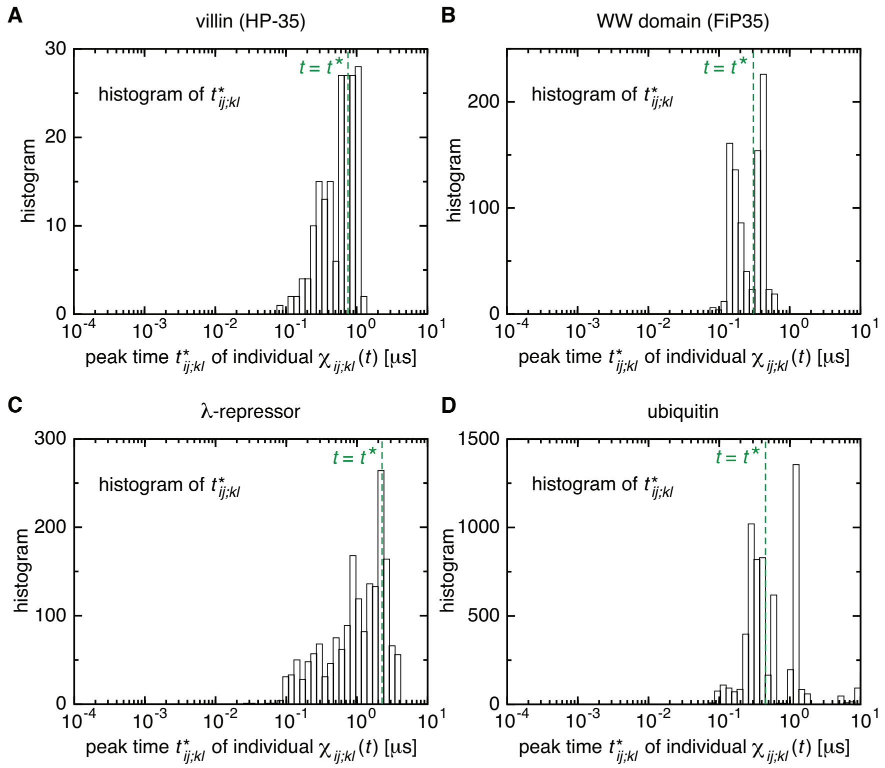


Figure S13: Dispersion of the peak times $t^*_{ij;kl}$ of individual $\chi_{ij;kl}(t)$ elements. (A) Histogram of the peak times $t^*_{ij;kl}$ of individual $\chi_{ij;kl}(t)$ elements of the villin headpiece subdomain on a logarithmic timescale. Only those elements for which $\chi_{ij;kl}(t^*_{ij;kl}) > 0.3$ were considered. The green vertical dashed line denotes the location of the average peak time $t^*$. (B–D) Corresponding results for the WW domain (B), $\lambda$-repressor (C), and ubiquitin (D).

Table S1: Protein systems studied in the present work

| | $N_{\mathrm{res}}{}^{a}$ | $T$ (K)$^{b}$ | $t_{\mathrm{sim}}$ ($\mu$s)$^{c}$ | $N_{\mathrm{TP}}{}^{d}$ | PDB entry$^{e}$ |
|---|---|---|---|---|---|
| villin (HP-35) | 35 | 345 | 398 | 12 | 1YRF |
| Nle/Nle mutant (HP-35) | 35 | 370 | 395 | 76 | 2F4K |
| WW domain (FiP35) | 35 | 395 | 600 | 23 | 2F21 |
| BBA | 28 | 325 | 325 | 13 | 1FME |
| NTL9 | 39 | 355 | 2,936 | 18 | 2HBA |
| BBL | 47 | 298 | 429 | 9 | 2WXC |
| protein G | 56 | 350 | 1,154 | 11 | 1MI0 |
| $\alpha$3D | 73 | 370 | 707 | 10 | 2A3D |
| $\lambda$-repressor | 80 | 350 | 643 | 11 | 1LMB |
| ubiquitin | 76 | 390 | 1,912 | 2 | 1UBQ |

[a] Number of amino acid residues. [b] Temperature at which the simulations were carried out. [c] Total simulation time (an aggregated simulation time for multiple simulation trajectories). [d] Number of folding transition paths. [e] PDB entry for the experimental structure used in defining native contacts.
